# The oblique plane deformity in slipped capital femoral epiphysis

**DOI:** 10.1007/s11832-014-0559-2

**Published:** 2014-02-20

**Authors:** Anthony Philip Cooper, Saif Salih, Carolyn Geddis, Patrick Foster, James A. Fernandes, Sanjeev S. Madan

**Affiliations:** 1 Department of Orthopaedic Surgery, Sheffield Children’s Hospital, Western Bank, Sheffield, S10 2TH UK; 2 Department of Orthopaedic Surgery, Northern General Hospital, Sheffield, S5 7AU UK; 3 Department of Orthopaedic Surgery, Musgrave Park Hospital, Stockman’s Lane, Belfast, BT9 7JB UK; 4 Department of Orthopaedic Surgery, Leeds General Infirmary, Great George Street, Leeds, LS1 3EX UK

**Keywords:** Slipped upper femoral epiphysis, Deformity, Classification

## Abstract

**Background:**

Slipped capital femoral epiphysis (SCFE) is commonly treated with in situ pinning. However, a severe slip may not be suitable for in situ pinning because the required screw trajectory is such that it risks perforating the posterior cortex and damaging the remaining blood supply to the capital epiphysis. In such cases, an anteriorly placed screw may also cause impingement. It is also possible to underestimate the severity of the slip using conventional radiographs. The aim of this study was to describe and evaluate a novel method for calculating the true deformity in SCFE and to assess the interobserver and intraobserver reliability of this technique.

**Methods:**

We selected 20 patients with varying severity of SCFE who presented to our institution. Cross-sectional imaging [either axial computed tomography (CT) scans or magnetic resonance imaging (MRI) scans] and anteroposterior (AP) pelvis radiographs were assessed by four reviewers with varying levels of experience on two occasions. The degree of slip on the axial image and on the AP pelvis radiographs were measured and, from this, the oblique plane deformity was calculated using the method as popularised by Paley. The intraclass correlation coefficient (ICC) was calculated to determine the interobserver and intraobserver reliabilities between and amongst the raters.

**Results:**

The interobserver reliability for the calculated oblique plane deformity in SCFE ICC was 0.947 [95 % confidence interval (CI) 0.90–0.98] and the intraobserver reliability for the calculated oblique plane deformity of individual raters ranged from 0.81 to 0.94. The deformity in the oblique plane was always greater than the deformity measured in the axial or the coronal plane alone.

**Conclusion:**

This method for calculating the true deformity in SCFE has excellent interobserver and intraobserver reliability and can be used to guide treatment options. This technique is a reliable and reproducible method for assessing the degree of deformity in SCFE. It may help orthopaedic surgeons with varying degrees of experience to identify which hips are suitable for in situ pinning and those which require surgical dislocation and anatomical reduction, given that plain radiographs in a single plane will underestimate the true deformity in the oblique plane.

**Level of evidence:**

Level II diagnostic study.

## Introduction

When assessing a patient with slipped capital femoral epiphysis (SCFE), several factors need to be addressed. These include the stability of the slip as defined by Loder et al. and the severity of the slip [1]. Most authors would agree that the treatment of choice for a mild or moderate slip is in situ pinning; however, controversy remains regarding the treatment of severe slips [2]. Indeed, there are multiple methods for defining a severe slip, and these are based upon plain radiographs [3]. There is evidence that an unstable slip should be treated within 24 h [4]; therefore, any method for assessing the severity of slip should be readily accessible, reliable and acceptable to the patient. It is important to identify a severe slip, as it may not be suitable for in situ pinning because the trajectory required of the screw is such that it risks perforating the posterior cortex and damaging the remaining blood supply to the capital epiphysis [5]. In such cases, an anteriorly placed screw may also cause impingement of the screw head on the pelvis [6]. It is also possible to incorrectly estimate the severity of the slip using conventional radiographs [1]. Furthermore, the more severe the slip, the greater the resultant deformity at the head–neck junction.

Based on the anteroposterior (AP) radiograph, Wilson defined a severe slip as one with a slip greater than half the metaphyseal diameter [7]. The Southwick angle is measured on the frog lateral radiograph. It is the difference in the head–shaft angle between the slip and non-slipped side. A slip of less than 30° is a “mild” slip using this method of measurement. A slip of greater than 50° is considered a severe slip [3]. These methods of classification are only based on one plane, either the coronal plane (AP radiograph) or the sagittal plane (lateral radiograph). However, the common deformity in SCFE is a combination of rotational torsion, varus (but valgus is possible) and posterior angulation. Therefore, the true direction of the deformity is oblique. Furthermore, the true magnitude in the oblique plane will be higher than that measured in the coronal or sagittal plane. Paley has popularised the concept that, if the magnitude of angulation is known in two orthogonal planes, the true oblique plane deformity can be calculated either graphically or mathematically [8].

**Fig. 1:**
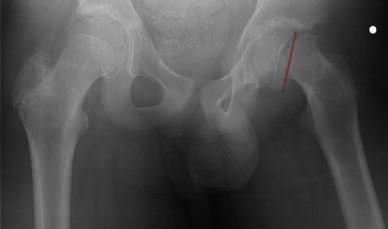
A line is drawn across the corners of the epiphysis on an anteroposterior (AP) radiograph of the pelvis

**Fig. 2:**
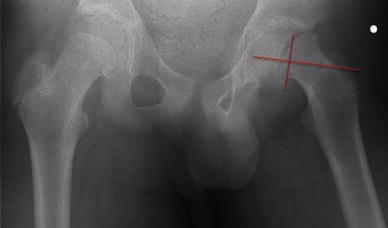
A second line is drawn perpendicular to the midpoint of the first line

In order to assess the true deformity in the oblique plane, we use a method which requires an axial cross-sectional image (transverse plane) and an image of the hip in the AP position (coronal plane). Using these two images, the deformity can be calculated in the oblique plane. The head–neck angle is measured from the AP pelvis film to give angle *x* (Fig. 1). The head–neck angle is measured from the axial computed tomography (CT) scan to give angle *y* (Fig. 2). From this. the oblique plane deformity angle *z* can be calculated.

To the best of our knowledge this technique has not been applied in this fashion previously. The technique for measuring angles *x* and *y* is as follows. Angle *x* is taken from the AP pelvis radiograph.

**Fig. 3:**
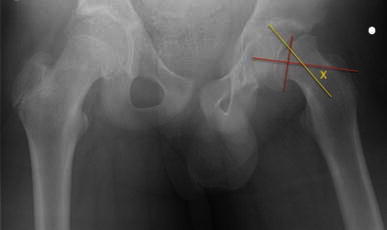
A third line is drawn along the axis of the femoral neck. The angle between the second and third lines is angle *x*, or the deformity in the coronal plane

**Fig. 4:**
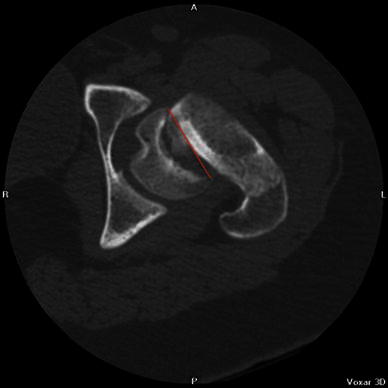
A line is drawn across the corners of the epiphysis on an axial computed tomography (CT) slice of the hip

**Fig. 5:**
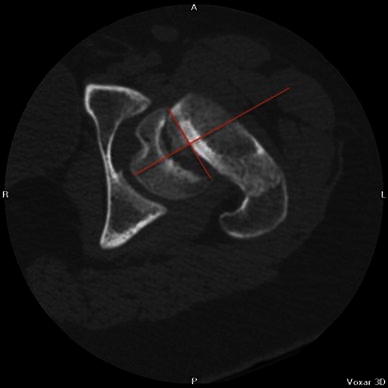
A second line is drawn perpendicular to the midpoint of the first line

**Fig. 6:**
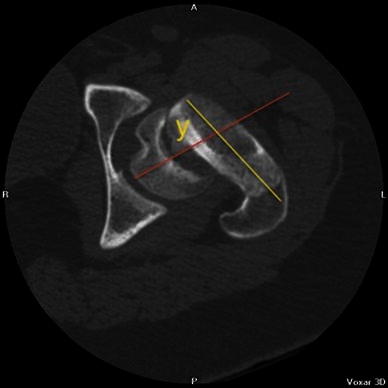
A third line is drawn along the axis of the femoral neck. The angle between the second and third lines is angle *y*, or the deformity in the transverse plane

The method of calculating the oblique plane deformity is explained in Figs. 1, 2, 3, 4, 5 and 6.

**Fig. 7:**
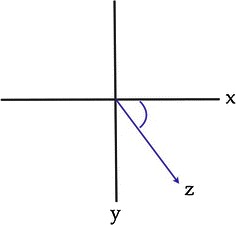
Graphical representation of deformity, where *x* = coronal plane angulation, *y* = transverse plane angulation and *z* = oblique plane deformity

 From these values, the oblique plane deformity can be calculated using the formula *z* = arctan√(tan^2^*x*) + (tan^2^*y*), but it can be more simply extrapolated to the calculation of *x*^2^ = *y*^2^ + *z*^2^ by means of Pythagoras’ theorem (Fig. 7).

It is important to accurately calculate the true degree of deformity in order to plan surgical treatment. It is our practice to perform surgical dislocation and anatomical reduction of the hip in patients with a slip angle greater than 50° in the oblique plane, regardless of stability [9]. In patients with a slip angle of less than 50°, we perform in situ pinning. If the patient is symptomatic, arthroscopy of the hip and debridement of the femoral head–neck offset is performed as a staged procedure.

## Materials and methods

We retrospectively analysed the records of all children referred to our unit with SCFE between July 2008 and January 2012 in order to identify 20 consecutive patients. Patients were selected if they met the inclusion criteria. The inclusion criteria were a diagnosis of SCFE, a well-oriented AP pelvis X-ray and the availability of axial imaging. As this was a sample of consecutive patients, we did not distinguish between the use of CT or magnetic resonance imaging (MRI) for the axial image. All images were obtained using a standardised imaging protocol. For the purpose of review, all patient information was removed and each hip assigned a study number. A total of 20 hips were included in the study. Approval of the clinical audit department was obtained.

Measurements were made independently by four different orthopaedic surgeons after agreeing the method to be used (one consultant, one fellow, two registrars). The observers received minimal training regarding the measurement methods. Training consisted of a demonstration of the angles to be calculated. No written training was received and no further training was given for the re-test element of the study. Observers with differing levels of experience were selected to see if the level of agreement varied among less experienced surgeons. The observers were blinded regarding patient history, physical examination findings and subsequent treatment.

For deformity in the coronal plane, the head–neck angle was measured from the AP radiograph. The deformity in the transverse plane was measured from the suitable axial image from the CT series (or MRI if not available). For each patient, the magnitude of the true or oblique plane deformity was calculated as previously described.

The intraclass correlation coefficient (ICC) was used to measure the intraobserver and interobserver reliabilities, with 1.0 being perfect agreement and 0 indicating agreement by chance alone. Excellent reliability was defined as an ICC >0.9, good 0.8–0.9 and fair 0.7–0.8. The intraobserver reliability was calculated for each variable (AP, axial, oblique plane) and the same images were re-examined by the same observers in order to calculate the intraobserver reliability for the oblique plane calculation between the first reading and the second reading.

Statistical analysis was performed using SPSS v19 (IBM, New York). The data were assessed for normality using both the Shapiro–Wilk test (with a *p*-value >0.05) and Q–Q plots of normality. Box and whisker plots of spread were calculated.

## Results

A total of 20 patients were identified, among which 17 had CT imaging available and three had MRI available only. There were 8 boys and 12 girls, with a mean age of 13 years (range 10–16 years). There were 12 unstable slips and 8 stable slips. The measurements for all patients for both the initial test and re-test can be found in the Appendix. Table 1 shows the mean values for the coronal plane angulation, transverse plane angulation and oblique plane calculation as measured on the initial test.

**Table 1:** Summary of the results of angular deformity measured on the initial test

Observer	Coronal angulation, mean (standard deviation)	Transverse angulation, mean (standard deviation)	Oblique plane angle, mean (standard deviation)
Registrar 1	34 (18.9)	67 (12.2)	75 (13.6)
Registrar 2	30 (20.2)	67 (13.4)	75 (16.9)
Fellow	37 (22.2)	66 (14.4)	78 (18.5)
Consultant	30 (18.6)	59 (17.9)	68 (21.2)

The interobserver reliability for all measurements performed during the first round of testing was excellent, with an ICC of 0.947 for the oblique plane calculation between all four observers (Table 2).

**Table 2:** Interobserver reliability

Variable	ICC	95 % CI	*p*-value
Coronal plane	0.964	0.930–0.984	<0.001
Axial plan	0.914	0.831–0.962	<0.001
Oblique plane	0.947	0.895–0.977	<0.001

The calculation of the ICC for the intraobserver reliability for individuals showed good to excellent correlation for all values, with a range from 0.800 to 0.968 (*p* < 0.001) for all values and a range from 0.814 to 0.941 (*p* < 0.001) in the oblique plane (Table 3).

**Table 3:** Intraobserver reliability between the first and second rounds of testing

Reviewer	Variable	ICC	95 % CI
Registrar 1	Coronal plane	0.968	0.92–0.987
	Axial plane	0.895	0.735–0.985
	Oblique plane	0.940	0.849–0.976
Registrar 2	Coronal plane	0.800	0.494–0.921
	Axial plane	0.934	0.834–0.974
	Oblique plane	0.814	0.529–0.926
Fellow	Coronal plane	0.943	0.855–0.977
	Axial plane	0.942	0.852–0.977
	Oblique plane	0.941	0.850–0.972
Consultant	Coronal plane	0.831	0.574–0.983
	Axial plane	0.865	0.659–0.947
	Oblique plane	0.881	0.700–0.953

**Fig. 8:**
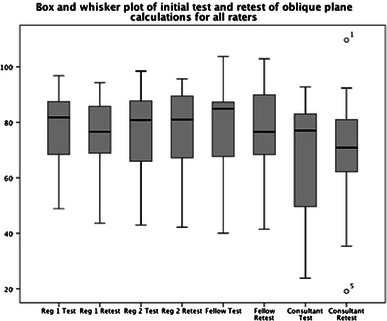
Box and whisker plot of the initial test and re-test of oblique plane calculations for all raters

Box and whisker plots of the spread of oblique plane calculations showed that there was no correlation between the seniority of the rater and the spread of values, with the lowest spread being from the two most junior raters (Fig. 8).

## Discussion

The reliability for our method of measuring the deformity in the coronal and axial planes can be classified as almost perfect agreement [10], with ICC values of 0.964 and 0.914, respectively. Our method of combining these two measurements for each patient has also been shown to have near perfect agreement (ICC 0.947) in terms of calculating the true magnitude of the slip in the 20 cases examined. This suggests that it could be reliably repeated in clinical practice in a more widespread manner. In our series, there was a wide variety of orthopaedic experience among the four assessors, which makes the reliability even more clinically relevant. In addition, there was substantial to almost prefect agreement in the intraobserver reliability for the test re-test results, despite a gap of 6 months between the two tests and no further training being given to the raters.

An AP radiograph in the supine position is easy to obtain and gives a reliable view. There are, however, certain technical difficulties associated with a lateral radiograph of the hip, particularly in a child with a painful SCFE. The standard frog lateral position can be painful and risks exacerbating the initial external rotation deformity [4]. The method described by Billing requires the femur to be externally rotated 90°, elevated 25° from the table, with the knee flexed 90°. This has been shown to be an accurate method of diagnosing a minor (particularly contralateral) slip, but has the same drawbacks as the frog lateral position for patients with possible major slips [11, 12]. In addition, the reliability of the Billing method has been questioned in cadaveric studies [13]. Furthermore, the more severe the deformity, the more restricted hip flexion and abduction becomes; thus, standardised lateral views become impossible. Other methods of obtaining a lateral view such as shoot-through or cross-table can be associated with significant soft-tissue shadow [14] (particularly in patients with a large body mass index, as is increasingly seen in these patients [15]) and may, therefore, be difficult to interpret [16]. For the purposes of this study, none of these “lateral” views can be guaranteed to give a truly orthogonal view to the AP radiograph, which is an essential prerequisite for using the geometrical method to calculate the magnitude of the oblique plane deformity [8].

These problems are overcome with cross-sectional imaging, such as CT or MRI. Both methods produce a true orthogonal view in the form of an axial image, which allows measurement of the posterior angulation and torsional component of the deformity. By combining this measurement with the displacement on the AP radiograph, the magnitude and direction of the true deformity in the oblique plane can be calculated. In our unit, we investigate children referred with possible moderate or major SCFE with both the AP radiograph and either or both of these methods. MRI was chosen in certain patients in order to assess for possible pre-operative avascular necrosis (AVN) [17]. For the purpose of measuring the slip angle, we do not believe that there is a significant difference between the two methods. However, the advantages of CT scanning are that it is more readily available, quicker to perform and has a lower cost than MRI scanning. However, the drawbacks are that the lifetime additional radiation risk of malignancy is increased from 1 in 100,000 for a pelvis X-ray to 1 in 10,000 for CT, thus increasing the risk from “very low risk” to “low risk”. This should be taken in the context of a 1 in 3 population risk of developing malignancy [18]. Furthermore, the radiology department at our institution is experienced in performing cross-sectional imaging for the assessment of rotational profiles. As such, it is able to provide the required cross-sectional imaging at the hip using targeted CT to minimise the radiation dose. Standard MRI sequences have not been found to be useful predictors of AVN in SCFE; however, the use of digital subtraction sequences has been shown to be promising in preliminary studies [9]. The advantage of determining whether there is pre-existing AVN is that it allows for greater confidence in predicting prognosis and planning further treatment.

Having calculated the oblique plane deformity, the management plan can then be made with more confidence. If the true magnitude is higher than first appreciated on initial radiographs, the decision may be made to perform open reduction or a corrective osteotomy. In addition, the degree of the true magnitude in the oblique plane may be used as a guide to prognosis.

We chose to use the AP pelvis rather than a coronal CT reconstruction to measure the deformity in the coronal plane because of our aim to identify a method which is readily available. The rationale for this was that some authors recommend urgent fixation of acute slips [4] and, as such, although it is often possible to obtain CT scans outside of normal hours, it is not always possible in our institution to obtain coronal re-formats. As such, we wished to evaluate whether our method was applicable using the readily available AP pelvis X-ray in order to provide a junior registrar with the means to rapidly assess whether a slip was suitable or not for in situ pinning.

There are several limitations to our study. The number of cases was relatively small; however, given that they all represented moderate or severe SCFE, there was not a significant heterogeneity of values in our samples.

Although it was not part of the original aims of the study, we found that several patients were seen on CT or MRI to have a marked curve to the femoral neck itself, rather than a straight neck and a sharp deformity at the physis (Fig. 3). These represented chronic slips with re-modelling. This is more in keeping with one of the early descriptions of SCFE by Ernst Muller in 1888 as “Schenkelhalsverbiegungen im Jungesalter”, meaning “bending of the femoral neck in adolescence” [19]. Knowledge of this deformity would be of obvious benefit before embarking on surgical intervention, as measurement of the displacement of the physis may be more difficult as re-modelling of the femoral head and neck occurs. Although re-modelling in chronic SCFE can cause retroflexion of the neck, the points on the femoral neck and head to measure the slip angle can be accurately delineated.

The magnitude of the mean angles in our series also gives important information. The mean deformity (66°, range 43–83) in the axial plane was twice that of the mean coronal plane deformity (33°, range 4–63). The mean oblique plane deformity was even higher at 75° (range 43–98). This shows that the true deformity can be significantly underestimated when relying on one image, particularly the AP radiograph. This study demonstrates that the true magnitude of the deformity in the oblique plane can be accurately and reliably measured with little inter- and intraobserver variability.

## Appendix

See Tables 4, 5 and 6.

**Table 4:** Coronal plane angulation values

Patient	Registrar 1	Registrar 2	Fellow	Consultant	Mean	SD
1	36	32	31	21	30	6.4
2	50	65	42	57	54	9.8
3	45	49	33	37	41	7.3
4	5	23	28	8	16	11.2
5	7	1	7	0	4	3.8
6	10	2	4	1	4	4.0
7	50	65	45	54	54	8.5
8	55	56	44	46	50	6.1
9	59	57	56	51	56	3.4
10	6	13	10	22	13	6.8
11	12	9	5	7	8	3.0
12	25	28	14	14	20	7.3
13	50	60	49	53	53	5.0
14	25	15	13	18	18	5.3
15	41	46	24	24	34	11.4
16	41	42	30	32	36	6.1
17	53	58	52	56	55	2.8
18	38^a^	25^a^	6^a^	30^a^	25^a^	13.6
19	18	35	36	21	28	9.3
20	57	69	62	63	63	4.9
Mean (1–20)	34	38	30	31	33	

^a^Valgus angulation

**Table 5:** Axial plane angulation values

Patient	Registrar 1	Registrar 2	Fellow	Consultant	Mean	SD
1	85	79	82	77	81	3.5
2	60	77	70	80	72	8.9
3	78	71	71	80	75	4.7
4	49	45	35	45	44	6.0
5	56	46	25	43	43	12.9
6	60	40	45	68	53	13.0
7	64	66	68	72	68	3.4
8	65	68	72	71	69	3.2
9	59	59	65	64	62	3.2
10	80	74	62	76	73	7.7
11	83	86	77	87	83	4.5
12	42	43	41	44	43	1.3
13	71	62	61	64	65	4.5
14	71	70	70	70	70	0.5
15	78	71	60	60	67	8.8
16	50	48	45	52	49	3.0
17	81	86	76	81	81	4.1
18	73	82	74	80	77	4.4
19	70	70	68	64	68	2.8
20	71	72	69	68	70	1.8
Mean (1–20)	67	66	62	67	66

**Table 6:** Oblique plane angulation values

Patient	Registrar 1	Registrar 2	Fellow	Consultant	Mean	SD
1	92	85	88	80	86	5.2
2	78	101	82	98	90	11.5
3	90	86	78	88	86	5.2
4	49	51	45	46	48	2.7
5	56	46	26	43	43	12.6
6	61	40	45	68	54	13.1
7	81	93	82	90	86	5.8
8	85	88	84	85	86	1.7
9	83	82	86	82	83	1.8
10	80	75	63	79	74	8.0
11	84	86	77	87	84	4.6
12	49	51	43	46	47	3.4
13	87	86	78	83	84	3.9
14	75	72	71	72	73	1.8
15	88	85	65	65	75	12.6
16	65	64	54	61	61	4.8
17	97	104	92	98	98	4.8
18	82	86	74	85	82	5.4
19	72	78	77	67	74	5.0
20	91	100	93	93	94	3.9
Mean (1–20)	77	78	70	76	75	
